# Melanoma-Derived Extracellular Vesicles: Focus on Their Proteome

**DOI:** 10.3390/proteomes7020021

**Published:** 2019-05-13

**Authors:** Magdalena Surman, Ewa Stępień, Małgorzata Przybyło

**Affiliations:** 1 Department of Glycoconjugate Biochemistry, Institute of Zoology and Biomedical Research, Jagiellonian University, Gronostajowa 9, 30-387 Krakow, Poland; magdalena.surman@doctoral.uj.edu.pl; 2 Department of Medical Physics, M. Smoluchowski Institute of Physics, Jagiellonian University, Łojasiewicza 11, 30-348 Krakow, Poland; e.stepien@uj.edu.pl

**Keywords:** proteomics, extracellular vesicles, melanoma, ectosomes, exosomes, cancer

## Abstract

Malignant melanoma is one of the most aggressive types of cancer, and its incidence is increasing rapidly each year. Despite the extensive research into improved diagnostic and treatment methods, early detection and disease constraint still present significant challenges. As successful isolation protocols have been developed, extracellular vesicles (EVs) have become the subject of extensive investigation in terms of their role in cancer progression and as a possible source of disease biomarkers. Besides functional studies, quantitative and qualitative proteomics have recently emerged as promising tools for the advancement of melanoma biomarkers. Nevertheless, the amount of data concerning the proteome of melanoma-derived EVs is still very limited. In this review we cover the current knowledge on protein content of melanoma-derived EVs, with a focus on their potential role in the development and progression of melanomas.

## 1. Introduction

Malignant melanoma is one of the most aggressive types of cancer, caused by neoplastic transformation of melanocytes (pigment-producing cells). Despite the prevalence of their cutaneous localization (>91%), primary melanoma tumors can also develop within the uveal tract or mucosal surfaces of the respiratory, gastrointestinal, and genitourinary tracts [1]. Global melanoma incidence is constantly rising, with approximately 350,000 new cases each year. Although melanoma incidence is 10-fold lower than the incidence of other skin cancers, its capacity to rapidly metastasize contributes to one of the highest mortality rates among all cancers, at about 60,000 deaths per year [2].

Despite the extensive research into improved diagnostic and treatment methods, early detection and disease constraint still present significant challenges. Melanoma markers routinely used in clinical practice for staging, prognostics, treatment selection, and follow-up include lactate dehydrogenase (LDH), S100B protein, melanoma inhibitory activity (MIA) protein, and *BRAFV^600E^* mutation [3]. These classic blood markers are constantly being revisited due to their insufficient diagnostic sensitivity and specificity. On the other hand, novel potential circulating biomarkers, such as cell-free DNA [4], microRNA [5], and circulating tumor cells [6], or a combination of autoantibodies [7], are being considered.

**Figure 1:**
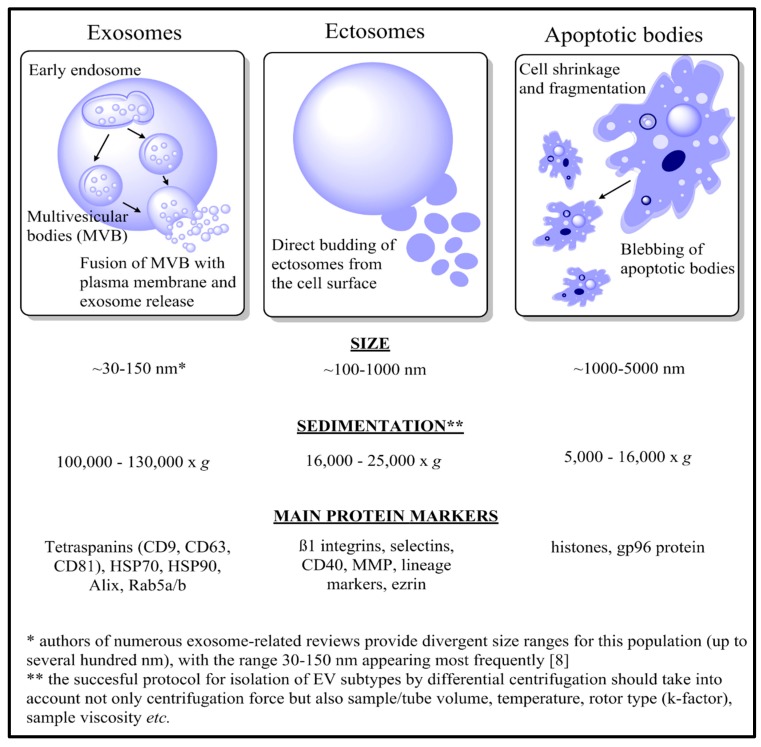
Brief characteristics of three major populations of extracellular vesicles: exosomes, ectosomes, and apoptotic bodies, in terms of their biogenesis, size, sedimentation, and main protein markers.

As successful protocols for the isolation of the extracellular vesicles (EVs) have been developed, they have become the subject of extensive investigation in terms of their role in cancer progression, and as a possible source of disease biomarkers. Viable cells predominantly release two types of EVs: ectosomes (also called microvesicles), which are directly shed from the cell surface, and exosomes, which are released after the fusion of multivesicular bodies (MVBs) with the plasma membrane. In the case of apoptotic cells, formation of apoptotic bodies during cell fragmentation is observed. Each apoptotic body contains an assortment of nuclear and cytoplasmic material, which may include intact organelles and their molecular components. The characteristics of these three major EV populations are presented in Figure 1 and reviewed thoroughly elsewhere [8].

Specific contents of EVs and their action towards the recipient cells depend on their molecular composition, which is determined by their origin cells. Proteins, lipids, and nucleic acids that are specific for a given cell type can be detected in respective EV populations, thus contributing to their prognostic and diagnostic value for individual health conditions, including different types of cancer [9,10,11]. Therefore, quantitative and qualitative proteomics of melanoma-derived EVs have recently emerged as promising tools for the advancement of melanoma biomarkers, but the amount of relevant data is still very limited. Nevertheless, the extensive proteomic analyses that have been carried out so far have confirmed the presence of melanoma-specific antigens and multiple oncogenic proteins in cargoes of melanoma-derived EVs. In this review we cover the current knowledge on protein content of melanoma-derived EVs, with a focus on their potential role in the development and progression of melanoma.

## 2. The Role of EVs in Melanoma Biology

All populations of EVs can be released by normal, as well as transformed, cancer cells. Upon their delivery to the recipient cells, EVs modulate a number of biological processes, including intercellular signaling, protein transport, cell proliferation, and cancer development. Melanoma-derived exosomes were shown to stimulate proliferation of cancer cells, to mediate activation of the epithelial-mesenchymal transition (EMT), and to induce pre-metastatic niche formation [12]. They also promote degradation of the extracellular matrix (ECM) by metalloproteinases (MMPs) [13], activate integrin signaling, and provide ligands for epidermal growth factor receptor (EGFR) and Notch receptor [12]. Furthermore, melanoma-derived exosomes modulate multiple functions of the immune system, most often by counteracting the anti-melanoma immune response [14]. They have also been shown to influence bone marrow progenitor cells toward a pro-metastatic phenotype through receptor tyrosine kinase MET [15] to regulate differentiation and maturation of antigen-presenting cells [16], to control survival and apoptosis of effector T-cells, and to control cytokine production and cytotoxicity of natural killer (NK) cells [12].

Although the vast majority of studies on melanoma-derived EVs have focused only on the exosome population, ectosomes may also facilitate disease development and progression. In the 1980s, different melanoma cell lines were shown to release ectosomes, which displayed the ability to boost metastatic potential when internalized by less invasive tumor cells [17]. More recent studies reported a two-fold higher number of ectosomes released in vitro by melanoma cells in comparison to normal melanocytes [18]. In addition, melanoma-derived ectosomes showed higher levels of tissue factor (TF), the main clotting initiator associated with cancer-related procoagulant state [18]. Melanoma-derived ectosomes may also promote tumor invasion and metastasis by transferring matrix-degrading metalloproteinases or their endogenous activators [19,20,21]. Finally, ectosomes may participate in tumor–stroma or tumor–immune cell interactions. Ectosome-mediated transformation of normal fibroblasts to tumor-associated fibroblasts has already been described in melanoma [22], as well as suppression of immune response by vesicle-associated Fas ligand (FasL) [23].

Even less is known about the role of apoptotic bodies in melanoma. However, in a sole study, apoptotic bodies released by mouse B16-F1 melanoma cells were isolated and revealed the highest procoagulant activity in in vitro coagulation assays in comparison to exosomes and ectosomes [24]. The procoagulant activity was attributed to the abundance of TF and phosphatidylserine in the apoptotic bodies under analysis. Additionally, mice inoculated with B16-F1 apoptotic bodies prior to inoculation with tumorigenic B16-F1 cells were protected from tumor development for up to 60 days, while a shorter tumor-free period was observed in mice immunized with B16-F1 exosomes or ectosomes. These results clearly demonstrated the involvement of melanoma-derived apoptotic bodies in procoagulant and immunogenic functions and their contribution to the prothrombotic state, as well as to anti-cancer immunity [24].

## 3. Isolation of Melanoma-Derived EVs for Proteomic Analysis

The credibility of proteomic data concerning any population of EVs depends primarily on the sample purity. Every single protocol for vesicle preparation and isolation should ideally lead to the obtainment of uncontaminated fractions of interest, i.e., within the predefined size and density range, free of cells, cellular debris, organelles, microorganisms, or aggregates, and characterized by a presence or absence of specific protein markers. Despite several decades of research and technological advancement, isolation of EVs still poses a significant challenge. Since the chosen isolation method may influence further analytical steps due to different chemical or physical factors used during the procedure, it should be decided based on the source of EVs (cell cultures or body fluids) and the specific downstream applications.

Regarding the sources of melanoma-derived EVs for proteomic studies, only conditioned culture media have been used so far. Except for one study using primary cell cultures established from biopsies of uveal melanoma tumors [25], other research works were performed using commercially available cutaneous melanoma cell lines representing various stages of the disease [24,26,27,28,29,30]. For maximum vesicle recovery, conditioned media should be collected from subconfluent (60–80%) melanoma cells [24,26,27,28,29,30]. Another good practice at the stage of melanoma cell culture is the use of fetal bovine (or calf) serum previously depleted of EVs [24,26,27,28,29,30], although bovine-specific protein sequences can be also eliminated during analysis of MS data. Alternatively, melanoma cells can be cultured for 24–48 h [25] in serum-free media, which is also thought to increase the amounts of released EVs. Regarding vesicle yield, expanded surface roller bottles for a maximal cell:supernatant ratio were recently used in proteomic studies of melanoma-derived exosomes [27].

The further preparation of EV samples for MS-based proteomic analysis requires a careful methodical approach, however the majority of known isolation methods can be successfully adapted. The most commonly used method for melanoma EV isolation is differential centrifugation, where centrifugal force determines the size of isolated particles. Besides centrifugal force (*g*-force), each protocol based solely or partially on centrifugation should include other factors that may to some degree influence EV sedimentation, i.e. rotor type, tube size, sample volume and viscosity, and temperature. Also, when changing the centrifuge, in many protocols centrifugation time is often adjusted by K-factor. However, Livshts et al. [31] demonstrated that such practice may be erroneous in the case of fixed-angle rotors. Instead they provided an equation to calculate the proportion of pelleted EVs of a given size based on applied centrifugation force, centrifugation time, and the sedimentation path lengths of a given fixed-angle or swinging bucket rotor. Using this method the centrifugation time required for complete sedimentation of the EVs of a given size can be easily calculated, and individual differential centrifugation protocols adequate for the centrifuge available can be developed.

Regardless of the differences at later stages, for the first centrifugation step low centrifugal forces, ranging from 300× *g* [25] up to 3,200× *g* [24,32], were routinely used to remove remaining cells and cell debris. Higher centrifugal forces were used to pellet larger vesicles (ectosomes and apoptotic bodies), however, their overlapping sizes (0.1–1 µm for ectosomes, 0.1–5 µm for apoptotic bodies) and densities makes their complete separation difficult. Muhsin-Sharafaldine et al. [24] used 25,000× *g* centrifugation to isolate both ectosomes and apoptotic bodies from in vitro culture of mouse B16-F1 melanoma cells. The only difference between isolation procedures was that supernatant for isolation of apoptotic bodies came from the cell culture treated with doxorubicin (pro-apoptotic agent), while vesicles from the untreated culture were considered as an ectosome population [24]. This approach can be justified by the fact that viable melanoma cells in a culture should not release apoptotic bodies. However, their presence in small amounts cannot be ruled out and should be analyzed with the use of exemplary apoptotic markers, such as histones or gp96 protein [33]. In addition, ectosomes and apoptotic bodies isolated according to the aforementioned protocol showed only a slight shift in terms of their diameter distribution, with modal values of approximately 250 nm for ectosomes and 350 nm for apoptotic bodies [24].

In contrast to high 25,000× *g* centrifugal force, another proteomic study on melanoma-derived EVs used centrifugation at only 10,000× *g* to pellet ectosomes released by mouse B16-F10 melanoma cells [29]. Such low centrifugal forces increase the risk of contamination with apoptotic bodies, but almost completely exclude the contamination with exosomes. In fact, quantitative analysis of proteomic data revealed a significant depletion of exosomal markers in the obtained melanoma ectosomes [29]. Taken together, these results suggest the need to carefully distinguish between melanoma-derived ectosomes and apoptotic bodies. Centrifugation steps of up to 16,000× *g* allow the separation of the population of larger apoptotic bodies, however, the use of 16,000–25,000× *g* will most likely produce a mixed population of ectosomes and smaller apoptotic bodies. The presence of the latter can be efficiently reduced if apoptotic processes in a cell culture are not over-induced.

For isolation of exosomes, intermediate steps up to 30,000× *g* can be used to remove cells, cell debris, and larger vesicles, followed by ultracentrifugation at 100,000–150,000× *g* to pellet exosomes. In proteomic studies on melanoma-derived exosomes, ultracentrifugation was often proceeded by ultrafiltration and concentration of the previously obtained supernatant with the use of 0.22 µm filters [24] or 100–500 kDa molecular weight cut-off membranes [27,29]. The ultracentrifugation step can be further modified by the addition of a density gradient, in which vesicles are separated according to their size and density. In this method, vesicles move in the gradient medium to the point where their density is equal to the density of the medium. Regarding commercially available kits, OptiPrep was used to isolate exosomes released by melanoma Mel501 cells on a 10–40% iodixanol gradient [30].

In another proteomic study on melanoma-derived EVs, the discontinuous step-gradient of 0.4–2.5 M sucrose in pH 7.4 HEPES (4-(2-hydroxyethyl)-1-piperazineethanesulfonic acid) buffer allowed for separation of two distinct exosome subpopulations. When a pre-cleaned and concentrated sample was loaded on top of a gradient, one exosome population was recovered, while loading the medium on the bottom of the tube and covering it with a sucrose gradient (the so-called bottom loading or upward displacement) resulted in separation of low density exosomes (1.12–1.19 g/mL) and high density exosomes (1.26–1.29 g/mL) [29]. Since ultracentrifugation can potentially affect exosome integrity, size-exclusion chromatography (SEC), which preserves all biophysical properties of EVs, was simultaneously used to prove that subpopulations of low- and high-density exosomes were not artifacts created during ultracentrifugation. In SEC, porous beads are used as a stationary phase. Large particles are eluted earlier from the column (they do not enter the pores), while smaller particles remain in the filtration column for longer (they are trapped in pores of the stationary phase). In the aforementioned study, melanoma-derived exosomes were isolated with the use of a Sephacryl column and then again successfully separated into two populations on a sucrose gradient [29]. Also, a gradient of low osmotic solution of Nycodenz was similarly used to separate exosomes. Another successful separation of low- and high-density exosomes ruled out the possibility that their different mobility within the sucrose gradient resulted solely from the damage to the exosomes caused by the hyperosmotic nature of the sucrose gradient [29]. In addition, after the use of different gradients for exosome isolation, it is necessary to remove any additional reagents from the sample before proceeding with the LC-MS protocol. For instance, melanoma-derived exosomes isolated on a sucrose gradient were dialyzed (10 kDa cut-off) against phosphate buffered saline (PBS) in order to remove sucrose, and later purified by the FASP (filter-aided sample preparation) method using ultrafiltration units with a 3 kDa cut-off [24].

Furthermore, isolation of exosomes does not necessarily require the ultracentrifugation step. As was shown by Xiao et al. [27], melanoma-derived exosomes can be isolated for proteomic studies with the use of additional precipitation agents. Cell culture media (previously depleted of cells, cell debris, and larger vesicles) were incubated with the commercially available Exoquick-TC polymeri c precipitation buffer, and exosomes were then pelleted by centrifugation at 10,000× *g* only [27]. Unlike other isolation methods, Exoquick-TC and similar kits are dedicated to smaller vesicles (approximately 60–180 nm in diameter), and thus still not applicable for isolation of ectosomes or apoptotic bodies. Furthermore, co-precipitation of protein aggregates is also commonly observed after the use of polymeric precipitation agents, and this can limit the identification of surface antigens in various analytical methods, for instance in flow cytometry.

To study the functional impact of melanoma-derived EVs present in patients’ body fluids on disease progression, isolation of EVs exclusively of melanoma origin from the total EV pool is necessary. Recently, Sharma et al. [34] developed an immunoaffinity-based method for exosome capture from the plasma of melanoma patients. Using mouse 763.74 antibody specific for the chondroitin sulfate proteoglycan 4 precursor (CSPG4) epitope uniquely expressed on melanoma cells, they successfully separated melanoma from non-melanoma exosomes confirmed by thorough analysis of their protein cargo. However, this method requires morphologically intact, non-aggregated exosomes, which carry the epitope of interest. Additionally, plasma-derived EVs are often coated by plasma proteins, which limit the access to surface antigens, thus the initial removal of plasma contaminants by SEC was proposed [34].

Finally, purity of an EV sample obtained with the use of a given isolation protocol should be analyzed in terms of vesicle size and the presence or absence of marker molecules. Methods that have been used for size characterization of melanoma-derived EVs include transmission electron microscopy (TEM) [24,27,28,29,32], nanoparticle tracking analysis (NTA) [28,29], and dynamic light scattering (DLS) [24], all of which also provide information on vesicle concentration. Phenotyping of melanoma EVs may be performed using flow cytometry [24] or western blot [27,28,29,30]. The commonly used markers for exosomes were tetraspanins (CD9, CD81), tumor susceptibility gene protein 101 (Tsg101), Alix, and HSP70. Other populations of melanoma-derived EVs were characterized by depletion or lower expression of exosomal markers, however, for apoptotic bodies histones or gp96 protein can be used [33], while ectosomes typically show enrichment in β1 integrins, CD40, TyA antigen, peptidase C1a, or Ezrin [35]. Such an approach follows the guidelines from the Minimal Information for Studies of Extracellular Vesicles 2018 (MISEV2018) [36], which does not propose molecular markers that could specifically characterize a single EV population; their presence should rather be analyzed in terms of their relative depletion or enrichment. Considering mechanisms of EV biogenesis, for each population membrane- or endosome-associated protein, markers should be provided, alongside exemplary cytosolic or secreted proteins that are expected to be recovered in isolated EVs. As a negative control the absence of nuclear, mitochondrial, or cytoskeletal proteins can be demonstrated for exosomes and ectosomes, while apoptotic bodies formed during cell fragmentation should contain such proteins. In terms of contamination, the presence of major components of non-EV co-isolated structures (e.g., aggregates of serum albumins or apolipoproteins, aggregates of uromodulin in urine, ribosomal proteins, etc.) should also be excluded.

## 4. Proteomic Techniques in Studies on Melanoma-Derived EVs

High throughput, specificity, and sensitivity have caused mass spectrometry to become the major tool used for identifying and characterizing molecular contents of EVs (mainly proteins), but also lipid and metabolite composition. A number of EV-oriented studies using qualitative and quantitative proteomics are constantly increasing, creating alternative analytical approaches, such as gel-based or shotgun proteomics. Novel, state-of-the-art methods allow identification of over 1,000 proteins in one EV sample, regardless of its source, i.e., cell cultures or body fluids [37].

Most of the latest research on EV proteome (summarized in Table 1) utilizes the common proteomic approach based on tryptic digestion of proteins, followed by separation of peptides by nano HPLC and their analysis by tandem mass spectrometry (MS/MS). In some cases the complexity of the EV sample is reduced prior to tryptic digestion by 1D or 2D SDS-PAGE (one/two-dimensional polyacrylamide gel electrophoresis in the presence of sodium dodecyl sulfate) separation of proteins. Lazar et al. [28] used 1D SDS-PAGE to separate exosomal proteins from 7 melanoma cell lines. The gels were routinely stained with Coomassie Brilliant G-250 Blue and each protein band was analyzed by mass spectrometry. Boussiadia et al. [30] also applied 1D SDS-PAGE to separate proteins from exosomes released by Mel501 melanoma cells, but the entire gel lines were cut into 25 slices, avoiding the loss of proteins (e.g., those located on the band margins, or not stained), which is inevitable when cutting out only stained bands.

In comparison to 1D SDS-PAGE, 2D SDS-PAGE includes an isoelectric focusing (IEF) step prior to separation of proteins by their molecular mass. It allows for separation and visualization of a much higher number of proteins from the complex samples, and for their more adequate quantitative analysis. Pardo et al. [38] used 2D SDS-PAGE before LC-MS/MS analysis of the conditioned media from the culture primary uveal melanoma UM-A cell line. Although no particular population of EVs was isolated, the study identified 133 proteins (the majority of them involved in melanoma biology) in excised gel spots. Analogous analyses of sera from uveal melanoma patients and healthy control revealed, based on different spot sizes, a differential expression of gp100 and cathepsin D that were later investigated as possible melanoma biomarkers [38].

To demonstrate differential expression of a protein within EV cargo, 2D DIGE (differential in-gel electrophoresis) can also be used. This method has an obvious advantage over traditional 2D SDS-PAGE, because it allows separation of up to three samples, stained differentially by fluorescent dyes (Cy3, Cy5, and Cy2) on one gel, thus overcoming in-gel differences. Xiao et al. [27] used 2D DIGE to compare protein profiles of exosomes released by normal HEMa-LP melanocytes and metastatic A375 melanoma cells. Finally, the major downfall of 2D techniques is that hydrophobic membrane proteins precipitate during IEF. Considering the membrane origin of EVs, a significant part of the EV proteome may not be observed and identified by MS if IEF is applied beforehand.

All of the aforementioned methods allowed for identification of up to 300 proteins in a single melanoma-derived EV sample. These numbers can be maximized with the use of a shotgun proteomics workflow, which has become a major tool, especially in large-scale proteomic profiling. The standard protocol starts with digestion of an intact (unseparated) protein mixture, thus the analysis is not limited by the modest separation efficiency of SDS-PAGE. Particularly, low-abundance proteins, aberrant proteins, as well as membrane proteins, can be more successfully identified. In studies based on shotgun proteomics, the number of proteins identified in melanoma-derived EV samples ranged from 539 [25] to even 2893 [29], proving the advantage of this approach.

Finally, shotgun proteomics can also be applied for quantitative analysis. Although no common methods of labeling, such as iTRAQ (isobaric tags for relative and absolute quantitation) or SILAC (stable isotope labeling in cell culture), have been applied to profile proteomes of melanoma-derived EVs so far, they proved useful in proteomic analysis of EVs from other types of cancer cells. For instance, Jeppesen et al. [39] used iTRAQ to identify proteins with differential expression in EVs from non-metastatic and metastatic bladder cancer cell lines, while Inder et al. [40,41] applied SILAC to compare proteome of EVs and of entire secretomes of prostate cancer PC3 cells. Alternatively, label-free quantitation can be performed based on spectral counting (SC), or spectral peak intensities (total ion current (TIC))-based approach. Boussadia et al. [30] used spectral counting quantification-exponentially-modified protein abundance index (emPAI) to compare proteomes of exosomes released be melanoma Mel501 cells cultured under normal and acidic pH.

## 5. Promising Results From In Vitro Studies on Melanoma-Derived EVs

The first study demonstrating the potential of global proteomic profiling of melanoma-derived EVs was performed by Mears et al. [26]. Protein profiles of exosomes purified from cell supernatants of two melanoma cell lines, MeWo and SK-MEL-28, were obtained using 1D PAGE alone and 2D PAGE, followed by MS analysis. Protein profiles obtained by these two different methods were highly congruent between exosomes but differed from the corresponding profiles of the total cell lysates. The 2D gels obtained for exosomes showed approximately 25% less protein spots in comparison to the total cell lysates, and only 45–50% of proteins were shared between corresponding lysates and exosomes, according to subsequent MS analysis. Forty-one exosomal proteins identified by MS included heat shock proteins, enzymes, and structural proteins with a variety of functions (according to Gene Ontology (GO) analysis), such as guanosine triphosphate (GTP)-binding, tetraspanin binding or apoptosis-related action. Interestingly, proteins present in whole-cell lysates that were absent in exosomes were mostly of mitochondrial, ER, Golgi, and lysosomal origin, which supports the endosomal origin of exosomes. This study was also the first to demonstrate the presence of several proteins in exosomes, namely ezrin, radixin, prostaglandin regulatory-like protein (PGRL), p120 catenin, syntaxin-binding proteins 1 and 2, Septin 2 (Nedd5), and tryptophan/aspartic acid (WD) repeat-containing protein 1 [26].

It would be useful clinically if proteomic profiling of melanoma-derived EVs allowed one to distinguish them from EVs released by non-transformed cells, and to indicate any differentially expressed proteins. For exosomes released by the highly metastatic A375 melanoma cell line and HEMa-LP normal melanocytes, 2D DIGE-MS analysis identified 11 differentially expressed proteins, including annexin A1, annexin A2, syntenin-1, and hyaluronan and proteoglycan link protein 1 (HAPLN1), all involved in angiogenesis, invasion, migration, and metastasis of melanoma cells [27]. In subsequent functional studies, normal melanocytes acquired invasive properties after incubation with melanoma-derived exosomes, suggesting that the specific protein cargo of tumor-derived exosomes may influence the process of carcinogenesis. Additionally, differential expression of 8 out of 11 exosomal proteins identified by MS correlated with expression levels of corresponding mRNA and upstream miRNAs that were also detected in melanoma- and melanocyte-derived exosomes [27].

Furthermore, the molecular content of EVs reflects the current state their origin cells. In that aspect proteomic profiling of melanoma-derived EVs may be used to indicate the proteins with differential expression, depending on the disease stage. Possibility of such application was preliminarily explored in studies using exosomes derived from 7 melanoma cell lines (3 non-tumorigenic, 2 tumorigenic, and 2 metastatic) [28]. Twenty-five percent of the proteins were shared between exosomes of all 7 origins, while the remaining 75% were present in exosomes released only by one or several cell lines. This suggested some degree of specificity and the dependence of the protein signature on the aggressiveness of a given cell line. Interestingly, according to gene ontology (GO) analysis categories of biological functions, such as cell motility, regulation of apoptosis, and angiogenesis, were significantly enriched only in the case of exosomes derived from metastatic melanoma cells [28].

It is generally accepted that besides the aforementioned exosomes, cells release at least two other types of EVs, i.e., ectosomes and apoptotic bodies. Due to their different biogenesis, these three populations have distinctive molecular contents and exert specific actions towards recipient cells. As the recent studies have shown, the proteomic approach can be applied alongside other methods to analyze phenotypic and functional differences between vesicle types originating from melanoma cells. MS-based profiling of exosomes, ectosomes, and apoptotic bodies derived from mouse B16-F1 melanoma in GO analysis revealed the enrichment of histones (H2A, H2B, H3.1, and H4) and heat shock proteins (HSPA5 and HSC71) in exosomes compared to ectosomes and apoptotic bodies. Additionally, based on ion scores, exosomes were enriched in tetraspanins CD9 and CD81, which belong to main exosomal markers [24]. Although the aforementioned enrichment of exosomes with histones should be considered as a possible contamination, Minimal Information for Studies of Extracellular Vesicles 2018 (MISEV2018) guidelines state that individual components from the endoplasmic reticulum, Golgi, mitochondrial, or nuclear compartments may be sorted into exosomes, especially in pathologic conditions that could affect protein incorporation [36].

Other studies considered the existence of subpopulations of exosomes that were obtained from mouse B16-F10 melanoma conditioned cell culture media using ultracentrifugation with sucrose density gradient [29]. Low- and high-density exosomes, alongside ectosome fraction for comparison, were subjected to MS analysis that allowed identification of 4,421 proteins in total. As many as 1,540 proteins were shared by all 3 populations, while 533, 354, and 110 proteins were identified exclusively in ectosomes, low-density exosomes, and high-density exosomes, respectively. Although exosomal markers (ALIX, CD9, CD63, and CD81) were detected in all subpopulations, their relative abundance was higher in both exosome subpopulations, as compared to ectosomes. The potential functional differences between the proteins identified across EV populations were revealed by GO analysis. Categories such as “oxidoreductases” and “dehydrogenases” were enriched in ectosomes and low-density exosomes, but not in high-density exosomes, suggesting the presence of mitochondrial components in ectosomes and low-density exosomes. In contrast, high-density exosomes showed higher enrichment within the categories of “translation”, “ribosomal proteins”, and “ribonucleoproteins”. Considering proteins identified in only one of EV subpopulations, low-density exosomes, were enriched in “small GTPase regulatory activity”- and “vesicle mediated transport”-related proteins, while high-density exosomes did not show the enrichment in any of GO categories, this suggests that low- and high-density exosomes represent two distinct subpopulations with unique protein compositions [29]. Despite the enrichment of high-density exosomes in ribonucleoproteins, questions may be raised as to whether this subpopulation originates from the remnants of ribosomal aggregates, e.g., from ruptured cells. Since ribosomes and mitochondria have similar densities to exosomes, they can be co-isolated, therefore the presence of exemplary mitochondrial or ribosomal markers should be analyzed in similar studies.

Finally, in vivo studies using cell culture-derived EVs come with certain limitations in terms of providing precise cellular conditions that would mimic the actual tumor microenvironment. For instance, the extracellular pH in the central region of tumors decreases because of lactate accumulation, while regular medium change in cell culture prevents the environment from getting acidic. Boussadia et al. [30] performed quantitative proteomic analysis of Mel501-derived exosomes obtained from cell cultures grown in standard conditions and in pH artificially lowered to pH 6.0. After 1D SDS-PAGE and in-gel trypsinization of the obtained bands 186 and 157, proteins were identified in control and pH 6.0 exosomes, respectively. Based on emPAI indices, the list of up- and down-regulated proteins in pH 6.0 exosomes was generated and subjected to functional annotation analysis using the KEGG (Kyoto Encyclopedia of Genes and Genomes) pathway database. Exosomes released under acidic conditions showed a significant enrichment within categories such as “proteoglycans”, “focal adhesion”, and “protein processing in endoplasmic reticulum”, while proteins involved in melanogenesis were down-regulated, suggesting the increased aggressiveness and reduced melanin content of Mel501 cells caused by pH 6.0. Subsequent GO analysis confirmed pH 6.0-associated abundance of proteins closely related to melanoma cell migration and with ability to influence melanoma malignancy. Enriched categories included cell-cell adhesion, leukocyte migration, regulation of cell shape, small GTPase mediated signal transduction, and epidermal growth factor receptor (EGFR) signaling pathways [30].

## 6. Clinical Significance of Proteomic Studies on Melanoma-Derived EVs

Despite the extensive research, the number of protein biomarkers with adequate clinical values in melanoma cells is still very limited. Development of quantitative proteomic studies on EVs isolated from different body fluids of melanoma patients holds promise for identification and validation of novel cancer cell specific biomarker candidates. Nevertheless, so far only a few comprehensive proteomic studies on melanoma secretomes have used patients’ samples. Firstly, the levels of established melanoma biomarkers, namely MIA, S100B, and tyrosinase-related protein 2 (TYRP2), were compared between exosomes isolated from the sera and total serum samples of stage IV melanoma patients and healthy controls. Higher concentrations of MIA and S100B were revealed in exosomes obtained from cancer-affected individuals, suggesting their potential diagnostic and prognostic utility [42]. Other studies showed higher levels of cathepsin D and gp100 in sera of patients with uveal melanoma compared to healthy volunteers’ sera [38]. Also, in a more recent study four melanocytic and fourteen primary uveal melanoma cell cultures were established from either cadaver (normal ocular melanocytes) or patient specimens (melanoma). The 2D SDS-PAGE LC-MS comparative analyses of their respective secretomes revealed 163 proteins differentially expressed between secretomes of melanoma and non-melanoma origin, with the levels of MIA and GDF15 (growth/differentiation factor 15 precursor protein) showing the most significant increase [25]. Although no particular population of EVs from sera was isolated in the last two studies mentioned above, it would be worth exploring whether these levels are reflected in sera-derived EVs. On the other hand, proteins that do not show significant enrichment or depletion in the entire sera or cell culture secretome may still be differentially expressed in particular populations of EVs isolated therefrom, and further evaluated as potential biomarkers.

Because of such limited data of clinical origin, the previously mentioned findings from the in vivo studies should be thoroughly reviewed. Future research could focus on biomarker candidates that were revealed in EVs released by melanoma cells, which could be used to direct the studies using patients’ samples in order to verify whether their differentiating levels are reflected at the organism level and are indeed clinically relevant. In that respect, apoptotic bodies released by mouse B16-F1 melanoma cells showed enrichment of the melanoma tumor-associated premelanosome protein (PMEL) [24]. Another proteomic study identified a set of proteins within exosome released by Mel501 cells, i.e., proto-oncogenes (HRAS, NRAS), tissue inhibitor of metalloproteinase 3 (TIMP3), heath shock protein isoforms (HSP90AB1, HSP90B1, HSPAIL, HSPA5), glucosidase II α subunit (GANAB), and actin-binding proteins (gelsolin, cofilin), all of which were previously found up-regulated in melanoma patients and associated with poor prognosis [30].

EVs are also carriers of established melanoma type-specific antigens. Using LC-MS, Lazar et al. [28] demonstrated the selective presence of melanoma-associated antigen 4, melanoma-associated antigen B2, and melanoma antigen recognized by T-cells in exosomes released by 7 different melanoma cell lines, suggesting that chosen exosomal proteins may prove useful in distinguishing between different melanoma subtypes. The same study also revealed the presence of proteins belonging to melanoma oncogenic pathways, including proto-oncogenes (NRAS, tyrosine-protein kinases Src, c-Met, and c-Kit), epidermal growth factor receptor (EGFR), and melanoma cell adhesion molecule (MCAM), which may all be involved in early melanoma development. Also, melanocyte-specific premelanosome protein (PMEL) was found by proteomic studies in exosomes derived from several melanoma cancer cell lines [43]. As access to deeper blood vessels is not achieved by non-transformed melanocytes unless vertical tumor growth occurs, circulating (plasma) melanocyte-specific exosomal protein markers could serve as an early indication of invasive melanoma growth. Finally, melanoma-derived exosomes were shown to contain a number of immunosuppressive proteins, such as galectins (LGALS1 and LGALS3) and 5′-nucleotidase (NT5E). Therefore, they may favor tumor escape from immune surveillance, which may contribute to, and explain, the ineffectiveness of some immunotherapies [28].

Unfortunately, so far only one clinical study (clinicaltrials.gov identifier: NCT02439008), exploring melanoma-derived exosomes as a source of potential protein biomarkers of radio- and immunotherapy response has been registered. This is in contrast to numerous proteomic studies in clinical settings, which follow different types of cancer. For instance, proteomic analysis of urinary EVs from prostate cancer patients revealed several markers for different cancer stages based on Gleason scores [44], while other studies identified changes in expression of 14 proteins correlated with glioblastoma invasiveness [45]. The diagnostic and prognostic potential of melanoma-derived EVs has also been demonstrated by a proteomic study [15]. A specific highly-expressed cargo (including Met oncoprotein) was identified in exosomes from metastatic B16-F10 melanoma cells in comparison to exosomes from less invasive B16-F1 cells. Functional in vivo studies show a direct contribution of exosomal Met in the education of bone marrow progenitor cells in establishing pre-metastatic niches in the lung and bone. Following this finding, exosomes from patients’ sera were also subjected to proteomic analysis and revealed a melanoma-specific exosomal protein signature that may potentially be used in disease staging and prognostics. With regard to breast and pancreatic cancer, proteomic analysis revealed specific protein profiles for exosomes derived from organotropic metastatic cancer cells [46]. When applied to melanoma EVs, such an approach may allow prediction of organ-specific sites of melanoma metastasis based on EV proteomic profiles.

Finally, EVs are also widely used as drug delivery systems (DDSs) [47]. Recently, attempts have been made to create artificial vesicles that would mimic the properties of natural EVs. The proteomic approach was recently used to compare protein cargo of natural neuroblastoma exosomes and exosome-mimetic vesicles derived from neuroblastoma cells by simple extrusion [48]. Proteomic data revealed a subset of proteins that were abundant in artificial vesicles in comparison to natural exosomes. Artificial vesicles contained proteins representing the whole parental cell proteome, whereas the exosomal protein set reflected their endosomal origin. This property of extrusion-made vesicles may alleviate the need for endosomal sorting of endogenous therapeutic proteins into exosomes, potentially also in melanoma.

## 7. Post-Transitional Modifications of Melanoma-Derived EVs: A Future Perspective

Proteomic techniques may also be applied to study post-translational modifications (PTMs) of proteins from melanoma-derived EVs. PTMs may influence several aspects of EV biology, including their biogenesis, protein sorting and localization, physical stability, and pathophysiologal action towards recipient cells. Glycosylation is the most common PMT and specific changes in glycosylation are considered the major hallmarks of cancer. Studies using Western Blot, flow cytometry, and lectin microarrays revealed the enrichment of melanoma-derived exosomes in high mannose, polylactosamine, α-2,6 sialic acid, and complex N-linked glycans [49,50], with the latter being directly involved in exosomal protein sorting [50]. Additionally, ectosomes were shown to be enriched mainly with fucose and complex type N-glycans with bisecting N-acetylglucosamine residues, which was demonstrated in our recent lectinomic study [32].

Proteomic techniques have not been applied to study PTMs in melanoma-derived EVs so far. However, glycosylation of EVs released by ovarian cancer cells has been analyzed by MS, which revealed the enrichment of heavily glycosylated tumor-associated galectin-3-binding protein G (LGALS3BP) in EVs [51]. Another MS-based study described the shift from high-mannose-type N-glycans to complex-type N-glycans in urinary EVs of patients with galactosemia [52]. Such changes in glycosylation with regards to particular disease states underline the importance of their analysis in the context of melanoma-derived EVs. In contrast to Western Blot, flow cytometry, or lectin microarray, which only confirm the presence of a given glycoepitope, MS-based analysis allows matching of specific glycan structures to proteins that possess them. Therefore, identification of particular proteins with aberrant glycosylation within EVs by MS should become a considerable direction in melanoma biomarker discovery.

Besides glycosylation, the presence of another PTMs in melanoma-derived EVs should be investigated. For instance, phosphorylation plays an important role in intercellular signaling and has already been addressed in the context of EVs by MS-based study. Gonzales et al. [53] identified 14 phosphoproteins in urinary exosomes from healthy donors, and multiple novel phosphorylation sites, including serine-811 in the thiazide-sensitive Na-Cl co-transporter protein. In an actual clinical context, label-free quantitative proteomic studies recently revealed 144 phosphoproteins that were differentially expressed in EVs from sera of breast cancer patients and healthy donors [54], indicating the need for similar studies on EVs derived from other types of cancer, including melanoma. Also, single proteomic studies reported the presence of ubiquitinated, sumoylated, oxidated, and myristoylated proteins in different types of EVs. The presence of these modifications may also affect protein sorting, EV aggregation, or their binding to the recipient cells, and has not been evaluated in melanoma-derived EVs so far [55].

## 8. Conclusions

A significant amount of data obtained from proteomic studies on melanoma-derived EVs provides a valuable background for identification of novel melanoma biomarkers and for their adjustment towards clinical use. Comprehensive proteomic characterization could be also used as a starting point for further studies on the molecular mechanisms leading to melanoma development and progression, and on the identification of the molecular pathways altered in melanoma subtypes, which could explain the various responses to the existing therapeutic treatments for this cancer. Nevertheless, the studies based on the proteomic analyses of melanoma-derived EVs only create the premise for the future prospective, large, independent, and multicenter trials that are necessary for the validation of any potential biomarker of cancer occurrence, tumor progression. or metastasis. However, the results of the already published studies are promising and suggest that these investigations should be continued in the future for melanoma, a disease for which effective biomarkers are lacking. Unfortunately, unlike well-explored methods for EV isolation and size characterization, the protocols for proteomic studies on melanoma-derived EVs are in the early stages of development. The few related studies that have been published so far used significantly different approaches, therefore, basic procedures for such experiments are still in need of normalization and verification.

## Figures and Tables

**Table 1:** The proteomic studies on melanoma-derived EVs (or secretome).

Source of EVs	Types of Isolated EVs	Methods of Isolation	Applied Proteomic Techniques	Number of Identified Proteins	Major Findings	Ref.
MeWo and SK-MEL-28 human melanoma cell lines	exosomes	0.1 µm filtration followed by ultracentrifugation at 100,000× *g*	2D SDS-PAGE-MS/MS (MALDI-TOF)	Forty-nine common protein spots in the exosome samples corresponding to 41 different proteins	p120 catenin, radixin, and immunoglobulin superfamily member 8 were identified in exosomes for the first time; mitochondrial and lysosomal proteins were significantly reduced in exosomes, confirming the endosomal origin of exosomes	[26]
MNT-1, G1 and Mel501 (non-tumorigenic), Daju and SK-MEL-28 (tumorigenic), A375 and 1205Lu (metastatic) human melanoma cell lines	exosomes	ultracentrifugation (100,000× *g*) and separation in sucrose (0.25–2.5 M) density gradient	1D SDS-PAGE followed by nano LC-MS/MS	between 486 and 632 depending on the cell line (517 on average); 917 unique proteins in all samples	exosomes from aggressive cells contained specific proteins involved in cell motility, angiogenesis, and immune response that were less-abundant or absent in exosomes from less-aggressive cells	[28]
Mel501 human melanoma cell line cultured in standard or acidic (pH 6.0) conditions	exosomes	ultracentrifugation (130,000× *g*) and separation in iodixanol (OptiPrep) gradient	1D SDS-PAGE followed by RPLC-MS/MS	three replicates for exosomes from pH 6.0: 212, 211, and 217 proteins; control: 194, 239, and 130 proteins	lower pH 6.0 modified exosome protein profile, causing up-regulation of more than 50% of the proteins	[30]
B16-F1 melanoma cell line (established from C57BL/6 mouse)	exosomes, ectosomes, apoptotic bodies	centrifugation at 25,000× *g* to pellet ectosomes and apoptotic bodies; the remaining supernatant was then filtered (0.22 μm) and exosomes were pelleted at 100,000× *g*; exosomes and apoptotic bodies were further purified by discontinuous sucrose cushion or linear sucrose gradient, respectively	uHPLC-MS (nanospray source of a LTQ Orbitrap XL)	553 proteins common to all populations	procoagulant proteins were more abundant in ectosomes and apoptotic bodies than in exosomes, with tissue factor (and lipid-phosphatidylserine) critical for procoagulant activity	[24]
B16F10 mouse melanoma cell line	exosomes, ectosomes	10,000× g to pellet ectosomes; exosomes were then pelleted from the remaining supernatant at 110,000× *g*; sucrose density and Nycodenz gradients were also applied to further separate exosomes; independent isolation of exosomes was performed using size exclusion chromatography (SEC)	nanoLC-MS/MS	a total of 4421 proteins; 1540 proteins common to all populations, and 533, 354, and 110 proteins were identified exclusively in ectosomes and low- and high-density exosomes, respectively	bottom-loading (instead of top-loading) of exosomes on sucrose density and Nycodenz gradients resulted in separation of high- and low-density exosomes displaying distinct protein profiles	[29]
melanoma A375 and normal melanocytic HEMa-LP cell lines	exosomes	500 kDa cut-off ultrafiltration followed by ultracentrifugation at 100,000× *g*, Exoquick-TC precipitation	2D DIGE-LC-MS/MS	total number not provided, 114 protein spots detected	differential expression of annexin A1, annexin A2, syntenin-1, and hyaluronian and proteoglycan link protein 1 between melanocytes and melanoma exosomes	[27]
primary cell cultures established from 14 original tumor specimens of uveal melanoma patients	not isolated, entire secretome was analyzed	-	label-free nanoLC-MS/MS	total of 1843 proteins (758 with at least 3 unique peptides)	a subsets of 83 up-regulated and 80 down-regulated proteins in uveal melanoma secretome	[38]
sera of uveal melanoma patients and healthy controls	not isolated, entire secretome was analyzed	-	2D SDS-PAGE- LS-MS (CapLC system coupled to a Q-TOF spectrometer)	133 (on average)	cathepsin D and gp100 protein levels increased in sera of uveal melanoma patients	[25]
